# Partitioning variance in immune traits in a zooplankton host—Fungal parasite system

**DOI:** 10.1002/ece3.9640

**Published:** 2022-12-19

**Authors:** Grace H. Westphal, Tara E. Stewart Merrill

**Affiliations:** ^1^ School of Integrative Biology University of Illinois Urbana‐Champaign Champaign Illinois USA; ^2^ Department of Biological Science Florida State University Tallahassee Florida USA; ^3^ Coastal and Marine Laboratory Florida State University St. Teresa Florida USA

**Keywords:** aquatic, crustacean, genetic structure, host–microbe, infection

## Abstract

Host immune traits arise from both genetic and environmental sources of variation. When immune traits have a strong genetic basis, the presence and severity of disease in a population may influence the distribution of those traits. Our study addressed how two immune‐related traits (gut penetrability and the hemocyte response) are shaped by genetic and environmental sources of variation, and how the presence of a virulent disease altered the relative frequency of these traits in natural populations. *Daphnia dentifera* hosts were sampled from five Indiana lakes between June and December 2017 before and during epidemics of their fungal pathogen, *Metschnikowia bicuspidata*. Collected *Daphnia* were experimentally exposed to *Metschnikowia* and assayed for their gut penetrability, hemocyte response, and multi‐locus genotype. Mixed‐effects models were constructed to partition variance in immune traits between genetic and environmental sources. We then isolated the genetic sources to produce genotype‐specific estimates of immune traits for each multi‐locus genotype. Finally, we assessed the relative frequency and dynamics of genotypes during epidemics and asked whether genotypes with more robust immune responses increased in frequency during epidemics. Although genotype was an important source of variation for both gut penetrability and the hemocyte response, environmental factors (e.g., resource availability, *Metschnikowia* prevalence, and co‐infection) still explained a large portion of observed variation, suggesting a high degree of flexibility in *Daphnia* immune traits. Additionally, no significant associations were detected between a genotype's immune traits and its frequency in a population. Our study highlights the power of variance partitioning in understanding the factors driving variation in *Daphnia* traits and motivates further research on immunological flexibility and the ecological drivers of immune variation.

## Graphical abstract

We found that genetics shape a large amount of variation in key *Daphnia dentifera* immune traits, but environmental factors were also important, suggesting a high degree of flexibility in their immune traits. We also found evidence that *Daphnia* population genetic structure does not shift toward more resistant genotypes during epidemics, which counters laboratory‐based evidence of rapid evolution of resistance and highlights the importance of considering the role of plasticity and the presence of multiple selection pressures when searching for evidence of natural selection.

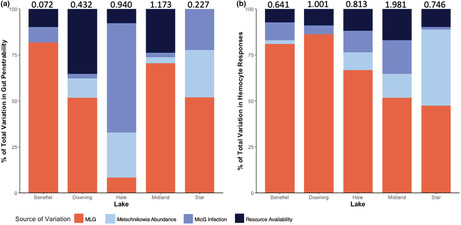

## 1 INTRODUCTION

When individuals within a population differ in their immune responses, disease has the power to influence the genetic structure of that population (Bonneaud et al., 2011; Voyles et al., 2018). For instance, mass mortality events in black abalone caused by a rickettsia‐like organism resulted in a substantial decrease in the genetic variation of abalone and shifted the mean immunity of the population toward increased resistance to the pathogen (Friedman et al., 2014). Likewise, epidemics of *Batrachochytrium dendrobatidis*, the fungal pathogen that causes chytridiomycosis in frogs, led to an increase in individuals with more effective anti‐fungal skin secretions (Voyles et al., 2018). While these examples show that disease may be a powerful driver of directional selection, the presence of pathogens can also maintain genetic diversity in a host population (Spurgin & Richardson, 2010). Understanding the genetic basis of immune traits, and how disease can shape their frequencies in populations, is thus a rich area of investigation.

In invertebrate species, many of which are ecologically and epidemiologically important, immune traits can be highly heritable and have high levels of additive genetic variation (Fellowes et al., 1998; Huang et al., 1999). Extensive research on model invertebrates (such as mosquitos and *D*. *melanogaster*) have shown that genes shape pathogen recognition, activation, and regulation of effector and signaling pathways, as well as immunological processes like myelinization, apoptosis, and autophagy (Kumar et al., 2018). This genetic basis for immunity suggests that the ability to resist parasites should vary among genotypes. This is certainly true for the snail *Biomphalaria glabrata*, in which susceptibility to infection with *Schistosoma mansoni* largely depends on the genotype of both the host and parasite (Richards & Shade, 1987). Additionally, laboratory‐reared populations of *Daphnia dentifera* also have different susceptibilities to infection with *Metschnikowia bicuspidata* based on clonal genotype (Hall et al., 2010).

Beyond underlying genetics, other intrinsic and extrinsic factors can affect immune traits and invertebrate–parasite interactions (Ben‐Ami, 2019). For example, young *Daphnia magna* tend to be more susceptible to infection by the bacterial parasite, *Pasteuria ramosa*, than older *Daphnia* (Izhar & Ben‐Ami, 2015). Immune traits, pathogen virulence, and host survivorship can also be affected by the quality and quantity of resources available to the host (Cressler et al., 2014; Hall, Knight et al., 2009). Likewise, even slight changes in the environment, such as subtle changes in temperature, can amplify the genetic differences between individuals causing changes in disease outcomes (Thomas & Blanford, 2003). For example, the survival of the mosquito *Anopheles stephensi* (a vector for malaria) varies with genotype, while parasite establishment and virulence, and the mounting of host defenses (like melanization) are altered by temperature and access to nutrients (Ferguson & Read, 2002; Koella & Sørense, 2002; Murdock et al., 2014). Finally, co‐infection with other parasites and pathogens can shape disease outcomes (Johnson & Hoverman, 2012). For example, infection with a microsporidian gut symbiont prevents infection by a virulent fungal pathogen in natural *Daphnia* populations (Rogalski et al., 2021). Taken together, these studies indicate that both genetics and the environment shape an individual's ability to resist disease. However, much of the current exploration into the genetics of immunity is largely dominated by studies of model organisms in highly controlled laboratory settings. Moving away from the laboratory and into natural populations is a growing frontier in biology and is imperative to understanding the basis of immunity in diverse and variable environments.

Identifying how much variation in immune traits is genetic and how much is environmental in naturally occurring populations—and what that means for their genetic structure during disease outbreaks—remains unknown for most systems. These questions represent key gaps in ecological immunology and disease ecology (Hawley & Altizer, 2011; Lazzaro & Little, 2009). Knowledge of the genetic versus plastic basis of immunity provides insights into how host interactions with parasites might shift over time and over changing environments (Gervasi et al., 2015). In addition, establishing whether disease can drive selection for infection‐resistant or more tolerant genotypes helps elucidate the capacity for population resilience and evolutionary rescue (Searle & Christie, 2021). We addressed these gaps using a model invertebrate disease interaction: the freshwater crustacean *Daphnia dentifera* and its fungal parasite *Metschnikowia bicuspidata*. This interaction provides an ideal system in which to partition natural variance in genetic and environmental factors contributing to susceptibility. First, methods have already been established to genotype *Daphnia* at six microsatellite markers (Fox, 2004; Holmes et al., 2016). Second, *Daphnia* in the field are parthenogenetic for most of the year, with little introduction of new genetic variation from sexual recombination. The repeated occurrence of genotypes across environmental conditions enables the partitioning of traits among genotypic and environmental sources of variation. Third, within populations of *Daphnia dentifera*, the *Metschnikowia* parasite has been observed to have limited detectable genetic variation, suggesting that genotype‐by‐genotype interactions are less important in this system than others (Searle et al., 2015; Shaw et al., 2021). Finally, the transparency of *Daphnia* allows researchers to visually assess infection and efficiently and effectively measure immune traits (Stewart Merrill et al., 2019; Stewart Merrill & Cáceres, 2018).

By linking genotype frequencies to immune traits during *Metschnikowia* epidemics, we explored whether clonal abundance is shaped by genetic resistance to disease. We partitioned variance in two key immune traits—gut penetrability and the hemocyte response—between genetic (multi‐locus genotype) and environmental sources of variation (resource availability, *Metschnikowia* prevalence, and co‐infection). We then isolated the genetic sources to produce genotype‐specific estimates of immune traits and explored how each genotype's immune traits were associated with its abundance during epidemics. Given that many *Daphnia* traits are influenced by both genetics and the environment (Allen et al., 2012; Deng, 1996; Tollrian & Leese, 2010), we predicted that the observed variance in both immune traits would have both environmental and genetic components. We also predicted that genotypes that were more resistant to infection (i.e., those possessing lower gut penetrability and higher hemocyte responses) would increase in relative abundance over the course of epidemics. We found that both genetics and the environment were important factors in determining the values of immune traits, but that clonal abundance during epidemics was not significantly associated with infection resistance. Our results raise exciting questions about the flexibility of *Daphnia* immunity and the drivers of genetic variation in immune traits.

## 2 METHODS

### 2.1 Study system

*Daphnia dentifera* is a freshwater crustacean zooplankter commonly found in Midwestern lakes in the United States of America. In *Daphnia dentifera*, parthenogenetic reproduction dominates for much of the year with sexual reproduction (producing dormant eggs) occurring in the fall (Cáceres & Tessier, 2004; Gowler et al., 2021). When dormant eggs hatch, genotypes that successfully establish in the water column contribute to genetic diversity (Crawford et al., 2015). For the rest of the ice‐free season, *Daphnia* are subject to fish predation, competition, and parasitism, which are all assumed to reduce clonal richness (Vanoverbeke & De Meester, 2010). However, field data demonstrating how these various selection pressures change population genetic structure remain rare.

*Metschnikowia bicuspidata* is an environmentally transmitted fungal parasite of *Daphnia* (Ebert, 2005). *Daphnia* are exposed to free‐floating fungal spores in the water column during suspension feeding. Infection occurs when fungal spores successfully enter the body cavity by penetrating the gut, thus gut penetrability is an important immune trait, with higher gut penetrability increasing the likelihood of infection (Stewart Merrill et al., 2019). If a spore penetrates the gut and enters the body cavity, it undergoes a series of developmental stages (Stewart Merrill & Cáceres, 2018). *Daphnia* can clear early developmental stages of *Metschnikowia* by mounting a hemocyte response, a second important immune trait, where greater hemocyte responses decrease the likelihood of infection (Stewart Merrill et al., 2019). *Daphnia* that are unable to prevent or clear infection with *Metschnikowia* die and release spores back into the water column (Ebert, 2005).

To analyze connections among disease dynamics, immunity traits, and clonal structure in *Daphnia*, we created new statistical models from field data previously published in Stewart Merrill et al. (2019), Rogalski et al. (2021), and Stewart Merrill et al. (2021). In brief, *Daphnia* were collected every 2 weeks from June to December 2017 from five lakes in central Indiana (Benefiel (*n* = 173), Downing (*n* = 184), Hale (*n* = 158), Midland (*n* = 190), and Star (*n* = 163)). Field‐collected *Daphnia* were experimentally exposed to *Metschnikowia* and assayed for their gut penetrability, hemocyte response, and multi‐locus genotype.

### 2.2 Host variables

Gut penetrability (probability that an infective *Metschnikowia* spore will penetrate the gut barrier) is the first line of defense for *Daphnia* and was measured for each individual by recording the number of spores that entered the body cavity versus the number of spores that attacked the gut epithelium but failed to enter the body cavity (Rogalski et al., 2021; Stewart Merrill et al., 2019, 2021). Hemocytes are used by *Daphnia* to clear early *Metschnikowia* infections and were quantified for each individual *Daphnia* by recording the total number of hemocytes observed attacking spores in the body cavity, as well as the total number of fungal spores in the body cavity (Stewart Merrill et al., 2019, 2021). The multi‐locus genotype (MLG) of each *Daphnia* was determined at six microsatellite markers using methods provided by Holmes et al. (2016). *Daphnia* sampled from the same lake that were identical at all six microsatellite markers were deemed the same MLG (Cristescu et al., 2006). It is possible that *Daphnia* that share the same microsatellite markers are not identical genotypes; that is, hidden genetic diversity can exist within an MLG. For simplicity, we denote individuals from the same lake that share the same MLG as a unique “genotype.”

### 2.3 Environmental variables

We focused on three core environmental variables that can shape immune traits and the outcome of host–parasite interactions: resource availability, co‐infection, and parasite abundance which in our case is *Metschnikowia* abundance (Hall, Knight, et al., 2009; Johnson & Hoverman, 2012; Reeson et al., 1998; Rogalski et al., 2021; Stewart Merrill et al., 2021). We quantified resource availability indirectly by calculating the mean number of eggs per egg‐bearing female. This index, the “egg ratio,” is a more sensitive index than traditional estimates for measuring primary production, such as chlorophyll *a* and total phosphorus, which can be poor predictors of the quality of available food (Tessier & Woodruff, 2002). Because egg production is resource dependent, a higher egg ratio is associated with a higher quality of available food (Tessier & Woodruff, 2002). We assessed co‐infection by focusing on the currently undescribed microbe designated as “MicG.” MicG is a microsporidian gut symbiont that alters the physiology of the *Daphnia* gut and reduces the gut's penetrability to *Metschnikowia* (Rogalski et al., 2021). In our study, “MicG infection,” refers to the prevalence of MicG within a population, where a higher prevalence should be associated with a higher concentration of MicG in the environment (Rogalski et al., 2021). We estimated *Metschnikowia* abundance by calculating the mean number of spores attacking the guts of wild‐caught *Daphnia* individuals (where an attacking spore is defined as a fungal spore that has attempted to penetrate the gut barrier) (Stewart Merrill et al., 2019). Attacking spores represents a higher risk of infection with *Metschnikowia* due to an increased concentration of *Metschnikowia* spores in the water column (Stewart Merrill et al., 2021). We estimated these three environmental variables for each lake‐by‐sampling event combination.

### 2.4 Statistical methodology

#### 2.4.1 Partitioning variance in immune traits

We were interested in how much of the observed variation in gut penetrability and hemocyte responses could be attributed to genetic (MLG) or environmental variation (resource availability, MicG infection, and *Metschnikowia* abundance). We constructed generalized linear mixed models for each lake to partition variance in the two traits. In our models, the only main effect was the intercept, and we incorporated genotype, resource availability, MicG infection, and *Metschnikowia* abundance as categorical random effects. We then attributed variance to each random effect by dividing the variance of the random effect by the total model variance (Crawley, 2013; Reeves et al., 2013; Stewart Merrill et al., 2022). Because the three environmental variables were continuous in their raw form, we needed to transform them into factors so that they would be suitable as random effects. We divided each environmental variable into “low” and “high” categories based on their median values. For example, if the median egg ratio within a lake was 3.5, all sampling events with egg ratios above 3.5 would be designated as “high resource availability” and all sampling events with values below or equal to 3.5 would be designated as “low resource availability.” With three environmental variables, each comprising two categories, our models allow us to partition variance among eight possible environmental combinations (the number of unique combinations is 3^2^ or 8). Variance partitioning models were constructed using the glmmTMB package in R (version 1.1.1) (Li et al., 2008; R Core Team, 2020).

To partition variance in gut penetrability, we constructed generalized linear mixed models with binomial error distributions for each of the five lakes. Successes (“1”) were defined as attacking spores that succeeded in penetrating the gut epithelial tissue, and failures (“0”) were defined as attacking spores that failed to penetrate the tissue (successes and failures were incorporated into the response variable using “cbind” in R). By considering the number of successes and failures for each *Daphnia*, this model can estimate gut penetrability on an individual basis, then partition its variation to the random effects of interest. To partition variance in the hemocyte response, we constructed generalized linear mixed models with Poisson error distributions for each of the five lakes. Because hemocytes represent count data, and possess strong skew, a Poisson error distribution was necessary to take into consideration the non‐parametric distribution. Because hemocyte counts increase with the number of spores infecting the body cavity (Stewart Merrill et al., 2019), we also included an offset term for the number of infecting spores observed.

#### 2.4.2 Estimating immune traits for each genotype

Our next question was whether epidemics shaped the relative abundance of genotypes depending on their susceptibility. To address this question, we first needed to isolate the genetic signal from the environmental noise. In other words, given that environmental conditions could influence the expression of immune traits, we sought a means to control for that environmental variation and extract genotype‐specific estimates of immune traits. To generate genotype‐specific estimates of gut penetrability and hemocyte responses, we constructed the same generalized linear mixed models as above (see “Section 2.4.1”), but in this case, incorporated genotype as a fixed effect, while keeping the categorical environmental variables as random effects (to control for variance attributable to the environment). Using the “predict” function on each model, we acquired model‐estimated values of gut penetrability and the hemocyte response for each genotype. Genotypes that have a gut penetrability of “1” have guts that are entirely penetrable to spores, where “0” represents guts that are completely impenetrable to spores. The hemocyte response output refers to the predicted number of hemocytes that would be recruited per spore.

We ran the models (above) for only frequently observed genotypes. By focusing on genotypes with larger representative sample sizes, we aimed to increase precision in estimating genotype‐specific immune traits and increase the likelihood that we would observe any changes in a genotype's abundance over time (see next section, “Section 2.4.3”). To that end, we generated estimates for the top 10 most common MLGs in each lake that were observed at least five times during the epidemic. We were unable to make predictions for hemocyte responses for *Daphnia* from Midland Lake, so we excluded these estimates from this and subsequent analyses. This challenge arose because only two genotypes were common (based on our criteria), and both possessed relatively impenetrable guts. Without successful spore penetration, spores with hemocytes in the body cavity do not exist, such that we could not model the hemocyte response.

#### 2.4.3 Clonal abundance

To address whether changes in clonal abundance during epidemics were associated with genotype‐specific immune traits, we quantified how the frequency of genotypes changed when disease spread through the population. We first calculated the relative frequency for each of the common genotypes from each lake (the top 10 most common that appeared at least five times in each lake during the epidemic) by dividing the number of individuals representing each genotype by the total number of genotyped individuals in the lake sampled on a given date. We arcsin transformed these frequencies because they represent proportional data. The epidemic timeframe from which relative frequency values were obtained ranged from the sampling date immediately prior to epidemic emergence (when the exposures leading to infection in the epidemic first occurred in each lake) through the course of the epidemic until sampling ceased (dates provided in Stewart Merrill et al., 2021). Epidemics were characterized by the growth in prevalence of late‐stage (transmitting) infections (Stewart Merrill et al., 2021). Using the dates bracketing epidemics allowed us to focus on when the parasite was being transmitted, and hence when selection from disease might have been occurring.

#### 2.4.4 Linking immune traits to clonal abundance

With genotype‐specific immune traits and clonal abundance patterns in hand, we could address our question of whether epidemics shaped the relative abundance of genotypes depending on their susceptibility. To explore patterns related to each genotype's relative frequency at the start of the epidemic, we regressed the relative abundance of each common genotype on sampling event with either genotype‐specific gut penetrability or genotype‐specific hemocyte response as a fixed effect. Importantly, in each of these models, we included an interaction term between sampling event and each genotype‐specific immune trait so that we could explore whether the change in a genotype's frequency over time was associated with its immunity. We also included lake as a random effect to control for variation between lakes. These models tell us two things: the fixed effect of the immune trait informs us of whether the relative abundance of each genotype during the epidemic was related to its immune traits. The interaction effect of these models tells us whether changes in clonal abundance during epidemics are associated with a genotype's susceptibility to infection, thus informing whether natural selection may have been acting on immune traits during the epidemic. Estimates for the hemocyte responses in Midland Lake were excluded from these analyses (see “Section 2.4.2”).

## 3 RESULTS

### 3.1 Partitioning variance in immune traits

**FIGURE 1:**
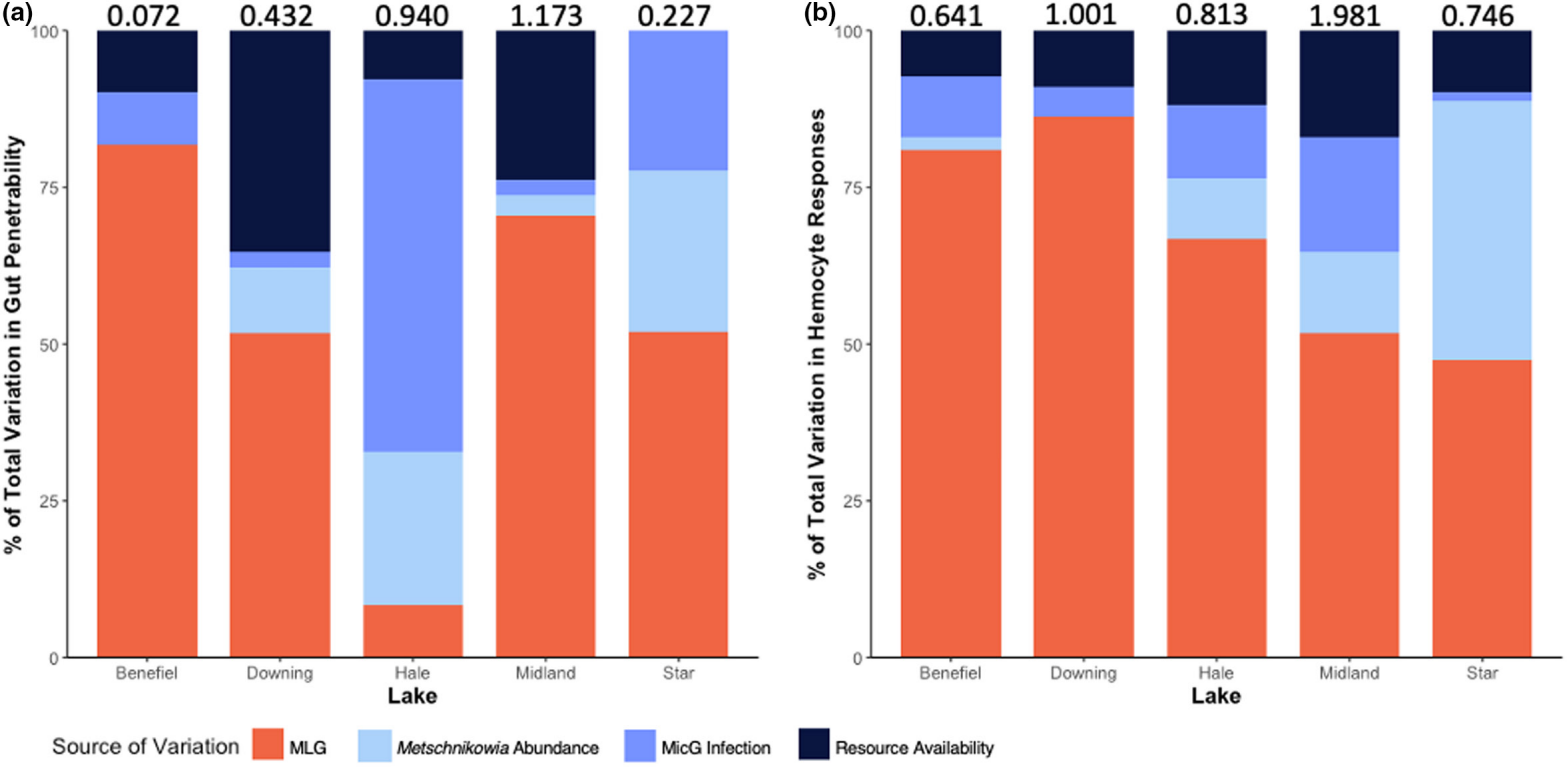
Genetic factors explain a large proportion, but not all, of variation in key Daphnia immune traits. (a) Genetic factors (orange bars, defined here as multi‐locus genotype, or “MLG”) explained 8–82% of the observed variation in gut penetrability (environmental factors represented by blue bars). A more penetrable gut increases the likelihood of infection with *Metschnikowia bicuspidata* spores. Therefore, in some lakes, genetic factors may play a more important role than environmental factors in determining an individual's likelihood of infection. (b) Genetic factors explained 47–86% of the observed variation in hemocyte responses (where greater hemocyte responses reduce the likelihood of infection with *M*. *bicuspidata*). Compared to gut penetrability, genetic factors had a more consistently influential role than the environment in determining an individual's hemocyte response. For both panels, total variation observed in each lake is placed over the bar. Non‐scaled variance partitioning is provided in the Appendix (Figure A1).

Lakes varied in the extent to which genetics and the environmental sources of variation that were measured (resource availability, MicG infection, and *Metschnikowia* abundance) shaped gut penetrability (Figure 1a). On average, genotype explained 52.9 ± 12.5% (SE) of the total variation observed in gut penetrability, peaking at 81.8% in Benefiel Lake and explaining only 8.4% of variation in Hale Lake. Hale Lake was the only lake in which genotype was not the largest single source of observed variation. The cumulative percentage of variation explained by the environmental factors was, on average, 47.1 ± 12.5% (SE).

For the hemocyte response, variance attributable to genotype accounted for nearly two‐thirds of the total variation observed, suggesting that genetics is more important than the environment for shaping *Daphnia* cellular immunity (Figure 1b). On average, across all lakes, genotype explained 66.6 ± 7.7% (SE) of total variation observed in the hemocyte response (the maximum was 86.3% in Downing Lake and the minimum was 47.4% in Star Lake). In Benefiel, Downing, Hale, and Midland Lakes, genotype was the single greatest source of observed variation in the hemocyte response. The cumulative percentage explained by environmental factors was on average 33.4 ± 7.7% (SE).

### 3.2 Estimating immune traits for each genotype

**FIGURE 2:**
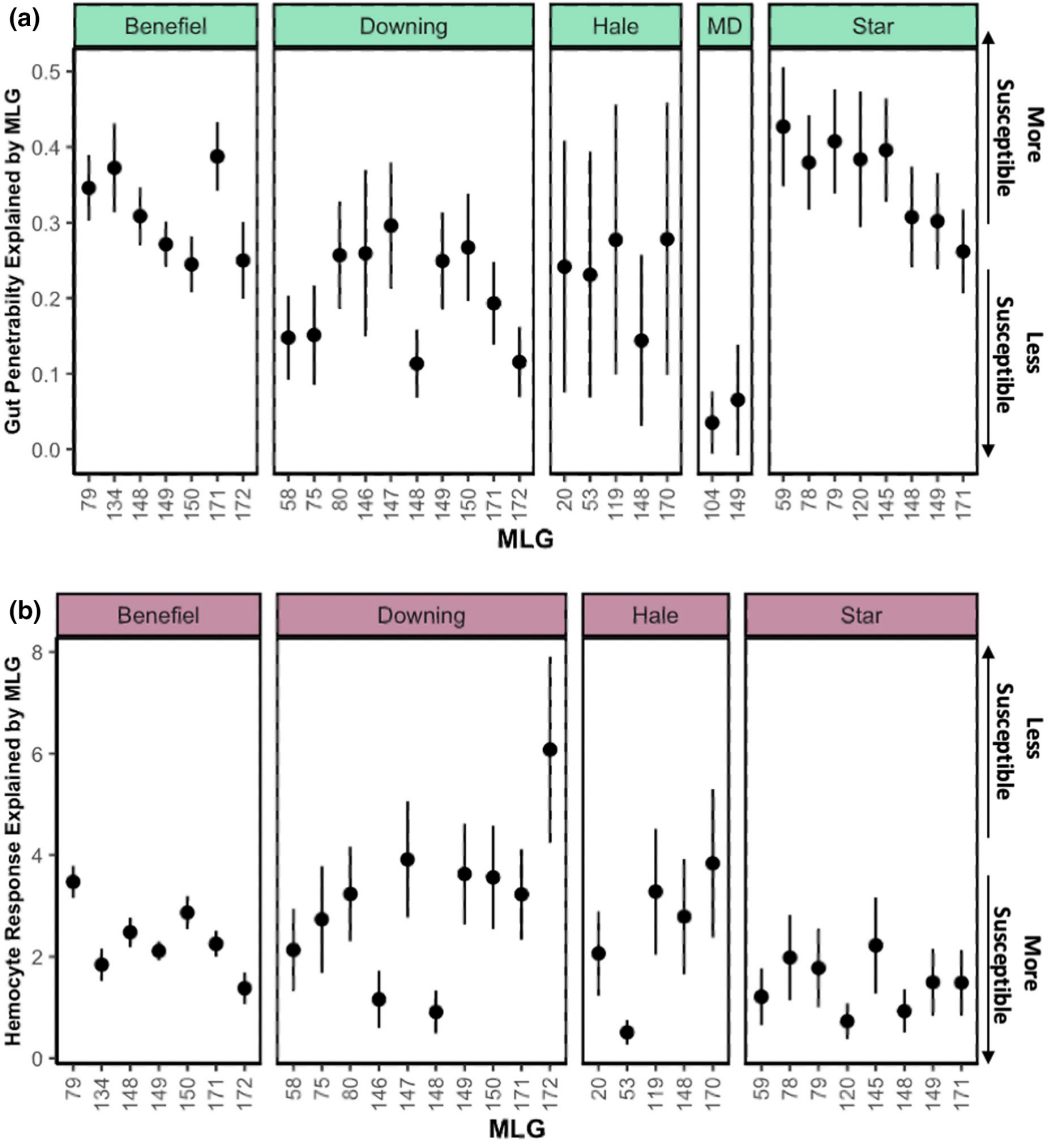
Genotypes within lakes vary in their immune traits. (a) Genotype‐specific gut penetrability (i.e., controlling for effects of different environments) ranged from highly impenetrable (i.e., near zero in Midland Lake) to moderately penetrable (35.8% in Star Lake). Gut penetrability varied among genotypes in Benefiel, Downing, Hale, and Star lakes. (b) Hemocyte responses varied among genotypes within each lake and ranged from approximately 0.51 (Hale; MLG 53) to 6.08 (Downing; MLG 172) hemocytes per spore, and 2.37 hemocytes per spore were recruited on average across all lakes. For both panels, each point on the chart (±SE) represents the model‐predicted value of a given immunity trait (gut penetrability and hemocyte response) for field‐collected *Daphnia* individuals that share the same MLG and were sampled from the same lake (for further details, see “Section 2.4.2”).

Genotype‐specific gut penetrability ranged from highly impenetrable (near‐zero in Midland Lake) to moderately penetrable (35.8% in Star Lake; Figure 2a). Within Benefiel, Downing, Hale, and Star lakes, there was significant variation in gut penetrability among genotypes (Figure 2a; Benefiel $\chi_{6,135}^{2}$ = 14.09, *p* = .028; Downing$\;{\;\chi }_{9,144}^{2}=22.77$, *p* = .007; Hale $\chi_{4,101}^{2}=$10.35, *p* = .035; Star $\chi_{7,110}^{2}=$19.13, *p* = .008). However, there was no significant difference in gut penetrability among the two genotypes in Midland Lake (Figure 2a; Midland $\chi_{1,24}^{2}=$2.17, *p* = .140). Within each of the lakes (with the exception of Midland which was excluded), there was significant variation in hemocyte responses among genotypes (Figure 2b; Benefiel, $\chi_{6,135}^{2}=$30.56, *p* < .001; Downing $\chi_{9,144}^{2}=$40.61, *p* < .0001; Hale $\chi_{4,101}^{2}=$53.12, *p* < .0001; Star $\chi_{7,110}^{2}=$42.04, *p* < .0001). Hemocyte responses ranged from 0 to 6 hemocytes per penetrating spore.

### 3.3 Linking immune traits to clonal abundance

**FIGURE 3:**
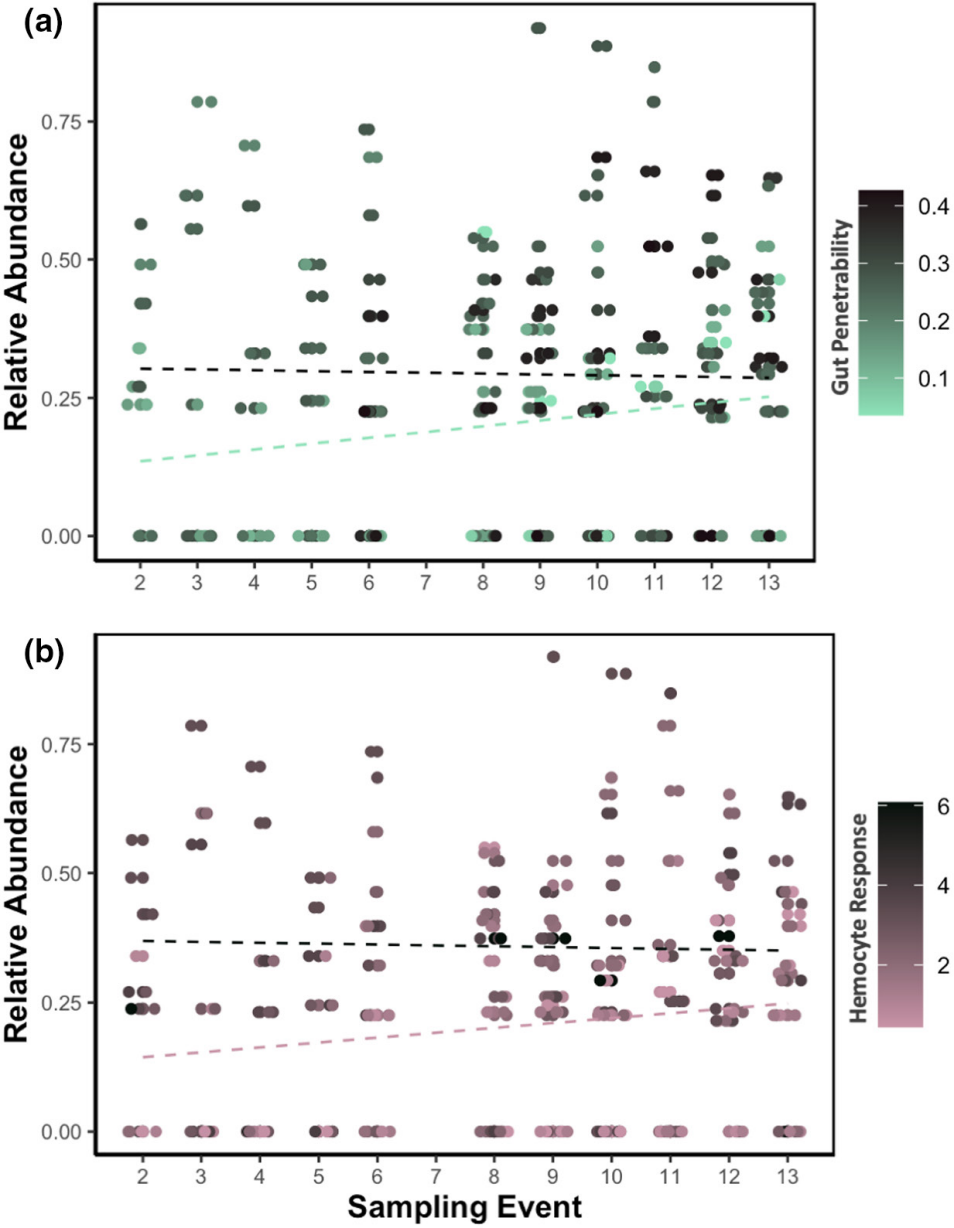
There are no relationships between immune trait estimates attributable to genotype and clonal abundance or changes in clonal abundance during epidemics. For both panels, relative abundance (on the y‐axis) is arcsin transformed, and each point represents a single clonal genotype and its relative abundance on each sampling event. (a) The change in a genotype's relative abundance during epidemics was not associated with its genotype‐specific gut penetrability. The teal line represents the predicted relative abundance during the epidemic of a genotype with the lowest observed gut penetrability (3.5%), and the black line represents the predicted relative abundance of a genotype with the most penetrable gut observed (42.7%). The proximity and similarity in slope between these lines suggest that differences in gut penetrability did not shape average relative abundance during the epidemics. (b) The change in a genotype's relative abundance during epidemics was not associated with its genotype‐specific gut penetrability. The pink line represents the predicted relative abundance during the epidemic of a genotype with the smallest observed hemocyte response (0.44 hemocytes/spore) and the black line represents the predicted relative abundance of a genotype with the largest observed hemocyte response (6.1 hemocytes/spore). The proximity and similarity in slope between these lines suggest that differences in hemocyte response did not shape average relative abundance during the epidemics.

We did not detect any significant associations between immune traits and the relative frequency of genotypes during epidemics. We observed no significant associations between each genotype's relative abundance during the epidemic and immune traits (Figure 3a; gut penetrability: *p* = .886; hemocyte response: *p* = .838). This suggests that the relative abundance of each genotype was not related to its gut penetrability or its hemocyte response. We also detected no significant interaction effect between genotype‐specific immune traits and sampling event, suggesting that the change in abundance of a genotype during an epidemic is not related to its susceptibility to infection (Figure 3b; gut penetrability‐by‐sampling event: *p* = .978; hemocyte response‐by‐sampling event: *p* = .800).

## 4 DISCUSSION

Disease can play a strong role in shaping population genetic structure (Friedman et al., 2014). We sought to estimate the extent to which host genotype versus environmental conditions shape immune traits in naturally occurring populations of *Daphnia*, and whether patterns of abundance during epidemics were related to *Daphnia* immune traits. We confirmed that *Daphnia* immune traits have a genetic basis, but were also variably shaped by resource availability, infection with gut symbionts (MicG infection), and environmental risk of infection (*Metschnikowia* abundance). In many cases, these environmental variables played a strong role in shaping *Daphnia* immune traits. Ultimately, we did not observe associations between patterns of clonal abundance and genotype‐specific immune traits. This may be because disease did not play an especially strong selective role (and other selection pressures may have acted simultaneously) or because the flexibility of the immune traits reduced each genotype's response to selection.

Through a statistical variance partitioning approach, we detected that both genotype and environmental conditions shape *Daphnia* immune traits in natural populations. More traditional variance partitioning analyses (using factorial laboratory experiments) have been applied to invertebrate immune traits with similar findings (Cotter et al., 2004; Lambrechts et al., 2006). Variance in infection intensity in a well‐known mosquito–malaria system (*Anopheles stephensi–Plasomidum yoeli yoelii*) was mostly dependent on environmental conditions, although a strong genetic signal was present (Lambrechts et al., 2006). Likewise, a study of Egyptian cotton leafworm *Spodopter littoralis* uncovered that both additive genetic effects and environmental variation explained variance within several of the components of hemolymph immune function (Cotter et al., 2004). Importantly, the extent to which genes and the environment interact to shape invertebrate immune phenotypes in the laboratory may differ from how they influence immune traits in natural systems, where sources of variation are diverse and continuous rather than filed into a few limited and discrete treatment groups (Lazzaro & Little, 2009). However, studies that assess the extent to which genes and the environment shape traits in natural systems are rare.

In the wild *Daphnia* populations we studied, genotype explained half of the variation in gut penetrability and nearly two‐thirds of the variation in the hemocyte response. This finding contrasts with a recent study on *Daphnia* immune traits, which found that genotype more strongly affected gut penetrability than environmental conditions (resource availability), whereas the hemocyte response was predominantly shaped by resource quantity (Cáceres & Stewart Merrill, 2022). Cáceres and Stewart Merrill (2022) performed their study under controlled laboratory conditions with three discrete categories of resources (low, medium, and high) and inoculated eight unique *Daphnia* genotypes with a single dose of *Metschnikowia* spores. But in the field, resource quantity and quality can vary dramatically over space and time (Scheffer et al., 1997), while parasite exposure can occur continuously and at varying levels (Cáceres et al., 2006). The difference between our findings suggests that clear relationships observed in laboratory studies can be complicated by high levels of variation and stochasticity present in the field.

Knowledge generated by laboratory studies can be critical for bridging the gap between highly controlled environments and natural systems. Of the three environmental variables we explored, two were informed by lab‐based research. First, we expected that resources would explain a large source of variation for both immune traits because the quality and quantity of food resources are known to shape interactions between *Daphnia* and *Metschnikowia* (Civitello et al., 2015; Hall, Knight et al., 2009; Hall, Simonis et al., 2009). Second, we expected that co‐infection by the gut symbiont, MicG, would be important in shaping gut penetrability given recent experimental results by Rogalski et al. (2021). Due to increased burden on the immune system, co‐infection is expected to weaken the host immune response (Jokela et al., 2000). Therefore, prior infection with MicG might alter the ability of *Daphnia* to mount a hemocyte response against *Metschnikowia*. While MicG infection represented a notable source of observed variance in gut penetrability in two of the lakes sampled (Hale and Star), this factor was less influential in the other three lakes. In those three lakes (Downing, Midland, and Benefiel), resource availability played a stronger role in shaping gut penetrability. In alignment with Rogalski et al. (2021), who found that resources and MicG infection shaped gut penetrability in opposing directions, we found that MicG infection and resources were approximately equal in shaping variation within the hemocyte response, both explaining the most variation in Midland and Hale lakes and playing less of a role in shaping hemocyte responses in Benefiel, Downing, and Star lakes. Co‐infection, it seems, is nearly as important as algal resources in explaining variation in both immune traits. By tying algal resources and co‐infection to immune traits, our study opens exciting opportunities to further study how these environmental pressures shape immunity and infection during epidemics.

The study is novel in exploring how risk of infection by *Metschnikowia* shaped immune traits. Because *Metschnikowia* does not show high levels of detectable genetic variation (Searle et al., 2015; Shaw et al., 2021), the risk *Daphnia* face should be proportional to the abundance of the pathogen in the environment. Some invertebrates with phenotypically plastic immune traits boost their immune responses when living in crowded conditions or in conditions that might lead to higher prevalence of disease (Reeson et al., 1998). However, we observed that the impact of *Metschnikowia* abundance on the variation present in immune traits was generally limited among lakes. Although *Metschnikowia* exposure has known suppressive effects on *Daphnia* foraging rates (Strauss et al., 2019), it may be that exposure to this fungal parasite has limited influence on *Daphnia* immunity.

During epidemics, strong selection imposed by disease can cause the genetic diversity of plant and animal populations to change (Friedman et al., 2014; Voyles et al., 2018). Our study evaluated whether a genotype's relative frequency during an epidemic was influenced by the immune traits of each genotype. We predicted that genotypes that were more susceptible to infection (those with high gut penetrability and low hemocyte responses) would be selected against during epidemics, and thus would decrease in abundance, or be rare, over the course of epidemics. Ultimately, we did not observe any associations between patterns of genotype abundance and immune traits during epidemics. This finding contrasts with the results of Duffy and Sivars‐Becker (2007), who observed that *Daphnia dentifera* in laboratory conditions can rapidly evolve decreased susceptibility over the course of *Metschnikowia* epidemics, curtailing pathogen spread in a theoretical model. The lack of association may be the result of disease playing a weaker selective role than expected, and the presence of other selection pressures within the environment. When multiple strong selection pressures are present within a given environment, the effect of one selective pressure may be minimized, altered, or obscured (Garcia et al., 2009; Postema et al., 2022; Sharma et al., 2020; Templeton & Shriner, 2004). Selection from the *Metschnikowia* epidemic was not the only selection pressure that *Daphnia* potentially faced during the time this data collection took place. In addition, *Daphnia* likely faced selection from predation (Leibold & Tessier, 1991), resources (Walsh et al., 2014), and even the presence of other parasites like *Pasteuria ramosa* (Decaestecker et al., 2007). We may also have not observed an association between abundance and immunity due to flexibility of immune traits which can reduce a genotype's response to selection. Evolutionary processes may not act as strongly when traits have low amounts of variation but high amounts of plasticity (Oostra et al., 2018), which our variance partitioning analyses suggested. Finally, we note that sexual reproduction likely did not contribute to the genotype frequencies observed because ephippia (indicative of sexual reproduction) were rare throughout the study period. Therefore, if disease is not a strong selective force and *Daphnia* immune traits are highly plastic, we may not expect disease to alter the genetic structure of *Daphnia* populations.

By taking the knowledge from laboratory studies about how environmental factors may affect *Daphnia* traits, we attempted to assess how these conclusions hold up in nature. Yet, there were limitations to our study design that decreased the probability of capturing the relationship between clonal abundance patterns and immune traits. First, we were unable to account for large and known sources of variation such as maternal effects and other within‐genotype sources of variation that can be controlled for with propagation of standardized maternal lines and sublines in the lab (Lynch & Walsh, 1998). Rather, our approach captured a breadth of variation from the natural environment, where we were only able to measure and analyze some of the sources of variation. In nature, *Daphnia* live in constantly fluctuating and variable conditions, and simulating an experiment that mimics such conditions would not be feasible. Another limitation of our study is that we were only able to capture the population dynamics of the most abundant genotypes because the sample size of less abundant genotypes was too low to apply and run the models used in this study. Therefore, we were unable to capture their immune traits or temporal patterns and potentially missed instances where less abundant genotypes were being selected for or against. More intensive sampling (to increase the sample size of all genotypes present), as well as establishing genetic markers for immune traits, would allow for inclusion of less represented genotypes in future studies.

In conclusion, our results suggest that both genetic and environmental variation are important for shaping *Daphnia's* susceptibility to infection with *Metschnikowia*. Our study raises several exciting questions. How does genotype shape immune responses in diverse ecological conditions? How plastic are *Daphnia* immune phenotypes? And is the selection pressure posed by disease strong or weak, or obscured by plasticity and other selective pressures present in the environment? Addressing the plasticity of *Daphnia* immune traits, and how genotype‐by‐environment interactions shape *Daphnia* immunity, will help us further understand the extent to which population genetic structure is shaped by disease.

## AUTHOR CONTRIBUTIONS

**Grace H. Westphal:** Conceptualization (lead); formal analysis (lead); visualization (lead); writing – original draft (lead). **Tara E. Stewart Merrill:** Conceptualization (supporting); data curation (lead); formal analysis (supporting); writing – review and editing (lead).

## CONFLICT OF INTEREST

The authors declare no competing interests.

## Supporting information


Appendix S1:

## Figures and Tables

**FIGURE A1:**
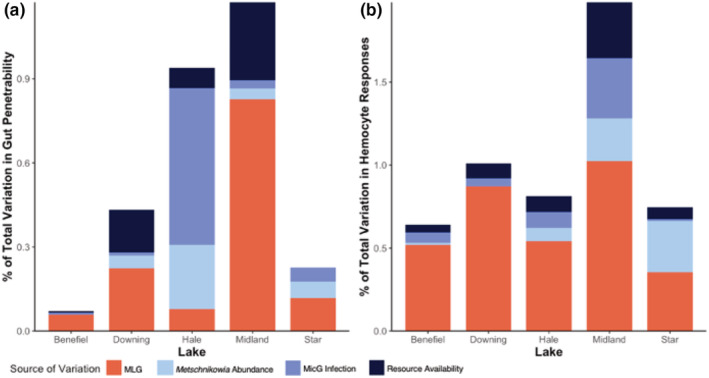
Non‐scaled variance partitioning. Genetic factors explained a large source, but not all, of variation in key immune traits, and the total amount of variance present in each immune trait differed between lakes. (a) Genetic factors (orange bars, defined here as multi‐locus genotype, or ‘MLG’) peaked at explaining 81.8% of variation in gut penetrability in Benefiel Lake and explaining only 8.4% of variation in Hale Lake. On average, genetic factors explained 52.9 ± 12.5% of the total variation in gut penetrability, whereas environmental factors (represented by the blue bars), explained on average 47.1 ± 12.5% of total variation. A more penetrable gut increases the likelihood of infection with *Metschnikowia bicuspidata* spores. Therefore, in some lakes, genetic factors may play a more important role than environmental factors in determining an individual's likelihood of infection. Total variance in the lakes ranged from 0.072 in Benefiel Lake to 1.173 in Midland Lake, suggesting that there are baseline differences among lakes in the amount of variation present in gut penetrability. (b) A greater hemocyte response reduces the likelihood of infection with *M. bicuspidata*. Genetic factors ranged from explaining 47.4% of variation in hemocyte responses in Star Lake, and 86.3% in Downing Lake. On average, across all lakes, genotype explained 66.6 ± 7.7% of total variation observed in the hemocyte response. Variance in the hemocyte response ranged from 0.641 in Benefiel Lake to 1.1981 in Midland Lake. Compared to gut penetrability, genetic factors had a more consistently influential role than the environment in determining an individual's hemocyte response.

## Data Availability

All data for *Daphnia* immune traits and environmental variables provided in the manuscript are publicly https://doi.org/10.5061/dryad.v15dv41ts. The multi‐locus genotypes associated with each *Daphnia* individual will be added to the dryad dataset, pending acceptance.
